# A nomogram prediction model for hungry bone syndrome in dialysis patients with secondary hyperparathyroidism after total parathyroidectomy

**DOI:** 10.1186/s40001-024-01801-y

**Published:** 2024-03-28

**Authors:** Dan Gao, Yali Liu, Wenpeng Cui, Xuehong Lu, Yan Lou

**Affiliations:** Department of Nephrology, The Second Hospital of Jilin University, 218 Ziqiang Street, Changchun, 130041 Jilin China

**Keywords:** Nomogram, Parathyroidectomy, Secondary hyperparathyroidism, Hungry bone syndrome, Risk factors

## Abstract

**Objective:**

Secondary hyperparathyroidism (SHPT) is a common complication of chronic kidney disease (CKD). Hungry bone syndrome (HBS) after parathyroidectomy (PTX) is a serious complication, which can lead to diarrhea, convulsion, arrhythmia and even death. This study was aimed to determine the risk factors for HBS after PTX in dialysis patients with SHPT and construct a nomogram prediction model to predict the incidence of postoperative complications.

**Methods:**

Clinical data were collected from 80 maintenance hemodialysis (MHD) patients with SHPT who received total PTX in the Second Hospital of Jilin University from January 2018 to September 2021. In line with the inclusion and exclusion criteria, totally 75 patients were finally enrolled for analysis. Patients were divided into two groups for retrospective analysis according to the severity of postoperative HBS, including HBS group and non-HBS (N-HBS) group. Univariate and multivariate logistic regression analyses were conducted to determine the risk factors for postoperative HBS. Afterwards, the receiver operating characteristic (ROC) curves were plotted based on the statistical analysis results, aiming to compare the prediction effects of different predicting factors. Finally, the nomogram was established to evaluate the occurrence probability of postoperative complications predicted by the risk factors.

**Results:**

Among the 75 patients, 32 had HBS (HBS group), while 43 did not have HBS (N-HBS group). Univariate analysis results indicated that, the preoperative intact parathyroid hormone (iPTH) and serum alkaline phosphatase (ALP) levels in HBS group were significantly higher than those in N-HBS group, while preoperative hemoglobin and preoperative albumin (Alb) levels were significantly lower than those in N-HBS group. As discovered by multivariate logistic regression analysis, preoperative iPTH (OR = 1.111, *P* = 0.029) and ALP (OR = 1.010, *P* < 0.001) were the independent risk factors for postoperative HBS. ROC curve analysis suggested that the area under the curve (AUC) values of these two indicators were 0.873 and 0.926, respectively (*P* < 0.0001). Subsequently, the nomogram model for predicting HBS was constructed. The model verification results indicated that the predicted values were basically consistent with the measured values, with the C-index of 0.943 (95% CI 0.892–0.994). Besides, the calibration curve was consistent with the ideal curve, demonstrating the favorable accuracy and discrimination of the model.

**Conclusions:**

Preoperative iPTH and preoperative ALP are the risk factors for postoperative HBS, which can be used to guide the early clinical intervention.

## Background

Secondary hyperparathyroidism (SHPT) is a common complication in patients with end-stage kidney disease (ESKD). SHPT refers to a group of clinical syndromes that involve the metabolic disturbance of calcium (Ca), phosphorus (P) and bone as well as multi-system injuries (including cardiovascular, neuromuscular and skin systems). This condition is induced by the hyperplasia and hypertrophy of parathyroid gland and the excessive secretion of intact parathyroid hormone (iPTH) due to factors like 1,25 (OH)_2_D_3_ reduction, hyperphosphatemia and hypocalcemia in the case of chronic kidney disease (CKD) that results in renal hypofunction [1–3]. Early SHPT can be partially controlled by regular hemodialysis (HD), low-P diet and drug therapy, but refractory SHPT still require early surgical intervention, otherwise, it will severely influence the quality of life and life span of the patients [4–6].

As is well known, persistent hypocalcemia will occur after parathyroidectomy (PTX) [4, 7]. With the increasingly in-depth understanding of this metabolic bone disease, such phenomenon of persistent hypocalcemia after PTX is named hungry bone syndrome (HBS). HBS is defined as the serum total Ca concentration sharply decreasing to lower than 2.1 mmol/l and/or the presence of long-term hypocalcemia for over 4 days after PTX [8]. Severe HBS may result in muscle spasm, epileptic seizure and even death [9]. Therefore, it is of importance to determine the high-risk factors for HBS after PTX, aiming to more closely monitor HBS after surgery [4].

Although HBS is common after PTX, research in this field is limited, and results from different studies are inconsistent [10–13]. In some studies, the surgical method is not unified, while the different surgical methods will greatly affect the postoperative serum Ca level. Some other studies investigate the risk factors for postoperative hypocalcemia, but they neither carry out model prediction and receiver operating characteristic (ROC) curve evaluation to predict the prediction accuracy, nor provide the cutoff values of risk factors.

PTH and ALP are currently the most concerned indicators related to HBS, the detection methods of PTH include serum iPTH and 1–84 amino acid full-length parathyroid hormone (wPTH), while iPTH is cheaper than wPTH, which is therefore more extensively applied in clinic. iPTH is an extremely vital regulatory factor for the bone reconstruction process, which can activate vitamin D3, enhance the renal hydroxylase activity, increase the number and activity of osteoclasts and thus elevate the serum Ca concentration. PTH at a physiological dose or small dose mainly functions to promote the osteoblast activity in bone, and enhance bone formation [14]. There are extensive sources of ALP in the human body, which exists in multiple cells and has 4 ALP isoenzymes. ALP is more widely distributed in human bones, and the serum ALP level increases when osteoblasts are activated.

This study aimed to investigate the independent risk factors for HBS after total PTX, the surgical method was unified, in other words, all patients received total PTX. Secondly, the ROC curves were plotted and the area under the curve (AUC) values and the cutoff values were calculated to determine the optimal cutoff values for diverse risk factors inducing adverse events. Thirdly, the incidence of postoperative complications was predicted using the nomogram prediction model and the model fitting effect was evaluated based on the calibration curve. Nomogram allows to visually display the risk factors affecting unfavorable outcome in a form of points, which contributes to screening the high-risk population, facilitates to formulate the targeted prevention and treatment measures, and assists in the early intervention of postoperative HBS and sufficient patient education to further guide clinical practice and effectively reduce the incidence of adverse events.

## Methods

### Objects of study

In this study, the clinical and biochemical data were retrospectively collected from maintenance HD (MHD) patients with SHPT who received PTX in the Second Hospital of Jilin University from January 2018 to August 2021. All patients signed the informed consent. This study was approved by the Ethics Committee of the Second Hospital of Jilin University.

### Inclusion and exclusion criteria

The patient inclusion criteria were as follows: ① patients receiving regular HD or regular peritoneal dialysis (PD); ② patients conforming to any one of the following indications of PTX [15]: a. persistently and significantly elevated PTH level (> 800 pg/ml); b. severe hyperphosphatemia and (or) hypercalcemia; c. severe clinical symptoms (including refractory skin itches, ectopic calcification, ostealgia, osteoporosis, height loss and fracture); d. the presence of at least 1 parathyroid gland hyperplasia lesion with the diameter of  > 1 cm or the maximal volume of 300 mm^2^ revealed on parathyroid gland color ultrasound; and e. tolerance to drugs like Ca-sensing receptor agonists, vitamin D and the analogues, which was not improved after medical treatment; and ③ patients receiving successful PTX, without ectopic parathyroid gland residue. The final pathological results indicated that, among the 75 patients, 11 had 3 parathyroid glands completely harvested, while the remaining 64 cases had 4 parathyroid glands completely dissected. Following the K/DOQI guidelines, this study adopted the minimal iPTH level  < 100 pg/ml within 7 days after surgery as the standard of successful surgery [16]. The patient exclusion criteria were shown below: ① patients taking Ca supplements or vitamin D analogues orally preoperatively after admission; ② patients whose parathyroid gland was incompletely resected and received repeated surgery; ③ patients with other concurrent severe diseases and complications; and ④ patients with poor treatment compliance.

### Preoperative preparation

For all the enrolled HD patients, the Ca concentration in the dialysis solution was 1.5 mmol/l, and the dialysis scheme was thrice a week, for 4 h each time. Preoperative localization diagnosis included parathyroid gland ultrasonography and sestamibi-single photon emission computed tomography (SPECT). All the ultrasonography examinations were completed by experienced physicians to assess the number, size, and location of parathyroid gland.

### Surgical method

All surgeries were performed by the professor from the Thyroid Surgery Department under general anesthesia. The patients received bilateral neck exploration (BNE) and total PTX, and the resected tissues were prepared into frozen sections to determine the pathological changes in all glands. Patients discovered with thyroid cancer in rapid intraoperative pathology received extended radical operation.

### Postoperative management

All HD patients received heparin-free HD treatment within 1 week after surgery; for patients with blood Ca level  < 2.1 mmol/l, the high-Ca dialysis solution (1.75 mmol/l) was used, while PD patients received PD peritoneal dialysis solution treatment. In week 1 after surgery, the biochemical indexes including serum Ca, P and ALP were re-examined in all patients every day in the morning; in addition, discomfort symptoms like hoarseness, neck hematoma, dyspnea, paresthesia, muscle spasms, tetany, circumoral numbness, and seizures were also observed. All patients were given Ca carbonate (element Ca, 9.6 g/day) and calcitriol (5 µg/day) immediately after surgery, and their dosages were adjusted daily based on the serum Ca levels of the patients. When the serum Ca level gradually decreased to below 1.8 mmol/l, intravenous infusion of Ca gluconate (rate, < element Ca 2 g/h) was added based on the basic oral dosages. When the serum Ca level became stable, intravenous infusion was gradually reduced and replaced with oral drugs. Patients were discharged when their serum Ca levels were stable at  > 2.1 mmol/l after completely withdrawing intravenous infusion of Ca gluconate.

### Data collection from objects of study

The sex, age, dialysis modality, dialysis duration, body mass index (BMI), number of parathyroid gland confirmed by parathyroid gland color ultrasound, number of parathyroid gland validated by parathyroid gland ECT, number of parathyroid gland verified by intraoperative pathology, serum iPTH, serum Ca, serum P, serum ALP, serum albumin (Alb), hemoglobin (Hb), thyroid stimulating hormone (TSH), and abdominal aortic calcification (AAC) score rated in the lumbar spine anteroposterior and lateral views of each eligible patient were collected. Then, patients were divided into HBS and N-HBS groups according to the postoperative HBS severity. HBS after surgery was defined as the minimum serum calcium level lower than 2.1 mmol/l (8.4 mg/dl) within 3 days postoperatively.

### Statistical analysis

SPSS 25.0 software was used for statistical analysis. For measurement data, the Kolmogorov–Smirnov method was adopted for normal distribution test, the normally distributed data were expressed as mean ± standard deviation (SD), and independent sample *t*-test (two-sided) was adopted for comparing the mean between groups. Meanwhile, the non-normally distributed data were described as inter-quartile range (IQR) and compared by rank sum test between groups. Enumeration data were expressed as frequency (percentage) and compared with Chi-square test or Fisher exact test between groups. Factors of *P* ≤ 0.05 were enrolled into univariate logistic regression analysis. Then, factors of significant difference upon univariate analysis were incorporated into multivariate logistic regression analysis. The odds ratios (ORs) and 95% confidence intervals (CIs) were calculated, and the prediction model was constructed. Furthermore, GraphPad 8.0 was used to plot the ROC curves to determine the optimal cutoff values of the influencing factors and to test the prediction performance of the logistic regression model. The R software studio (R version 4.1.0) was applied to construct the nomogram prediction model and to draw the nomogram. Afterwards, the Bootstrap method (repeated sampling of raw data) was used for internal verification of the nomogram model. *P* < 0.05 indicated statistical significance.

## Results

### Baseline information of the objects of study

Totally 80 SHPT cases were collected at our department in 44 months. After screening according to the inclusion and exclusion criteria, a total of 75 patients undergoing t-PTX were enrolled finally. Among the enrolled cases, there were 32 (42.67%) patients in HBS group and 43 (57.33%) in N-HBS group. The age of all patients, including 49 male (65.3%) and 23 female (34.7%) patients, ranged from 23 to 69 (average 46) years. In addition, 73 (97.3%) cases received HD, and 2 (2.7%) underwent PD, with the average dialysis duration of 8.19 years. Among the enrolled patients, 11 had 3 parathyroid glands resected, and the remaining 64 (85.3%) had 4 parathyroid glands resected. In addition, 7 (9.3%) patients had concurrent thyroid cancer. The preoperative Hb (*P* = 0.001) and preoperative Alb (*P* = 0.021) levels in HBS group were significantly lower than those in N-HBS group, while the preoperative ALP (*P* < 0.001) and preoperative iPTH (*P* < 0.001) levels in HBS group were significantly higher than those in N-HBS group. Differences in age, sex, dialysis modality, concurrent hypertension, concurrent diabetes, dialysis duration, BMI, number of parathyroid gland detected on color ultrasound, number of parathyroid gland detected on ECT, number of parathyroid gland detected based on intraoperative pathology, preoperative BNP, serum Ca, serum P, TSH and AAC score between two groups were not significant (*P* > 0.05) (Table 1).

**Table 1:** Baseline characteristics of all patients and possible predictors of postoperative HBS in patients with SHPT

Predictor	All patients (*n* = 75)	Non-HBS (*n* = 43)	HBS (*n* = 32)	Statistic	*P* value
Gender (male/female)				χ^2^ = 0.288	0.592
Male	49 (65.3%)	27 (62.8%)	22 (68.8%)		
Female	26 (34.7%)	16 (37.2%)	10 (31.3%)		
Dialysis modality				χ^2^ < 0.001	1.000
Hemodialysis	73 (97.3%)	42 (97.7%)	31 (96.9%)		
Peritoneal dialysis	2 (2.7%)	1 (2.3%)	1 (3.1%)		
Hypertension	69 (92.0%)	38 (88.4%)	31 (96.9%)	χ^2^ = 1.802	0.362
Diabetes	5 (6.7%)	2 (4.7%)	3 (9.4%)	χ^2^ = 0.649	0.731
Age (years)	46.49 ± 11.02	48.09 ± 11.79	44.34 ± 9.66	*t *= 1.469	0.146
Duration of dialysis (years)	8.0 (5,10)	8 (5,10)	9 (7,10)	*Z* = −1.169	0.242
BMI (kg/m^2^)	22.77 ± 3.71	23.20 ± 3.79	22.19 ± 3.57	*t *= 1.158	0.251
Frequency of color ultrasound	4 (3,4)	3 (2,4)	4 (3,4)	*Z* = −1.359	0.174
Frequency of ECT	4 (4,4)	4 (4,4)	4 (4,4)	*Z* = −0.475	0.635
Frequency of pathological examination	4 (4,4)	4 (4,4)	4 (4,4)	*Z* = −0.454	0.649
Preoperative Hb (g/l)	116.85 ± 18.88	123.19 ± 17.01	108.34 ± 18.12	*t* = 3.635	0.001
BNP (pg/ml)	156 (62,359)	119 (58,265)	249.5 (78.75,508.5)	*Z* = −2.105	0.035
Alb (g/l)	42.9 (40.3,44.6)	43.7 (42.0,45.2)	41.4 (38.1,43.4)	*Z* = −2.973	0.003
Serum ALP (U/l)	292 (144,545)	173 (118,287)	683.5 (354.3,974.8)	*Z* = −6.277	< 0.001
Serum calcium (mmol/l)	2.45 ± 0.26	2.50 ± 0.26	2.40 ± 0.25	*t* = 1.666	0.100
Serum phosphate (mmol/l)	2.04 (1.75,2.39)	2.05 (1.78,2.33)	2.00 (1.71,2.51)	*Z* = −0.021	0.983
iPTH (pg/ml)	2170 ± 990	1619 ± 668	2911 ± 864	*t* = −7.309	< 0.001
TSH (mlu/l)	1.59 (0.99,2.60)	1.45 (0.99,2.68)	1.64 (1.02,2.35)	*Z* = −0.107	0.375
AAC score	3 (0,15)	3 (0,14)	5 (0.25,16.5)	*Z* = −0.708	0.479

### Univariate logistic regression analysis on the incidence of HBS after t-PTX

Results of univariate logistic regression analysis indicated that, the occurrence of HBS after t-PTX was related to preoperative Hb, Alb, iPTH, and ALP levels (*P* < 0.05). Differences in age, sex, dialysis modality, concurrent hypertension, concurrent diabetes, dialysis duration, BMI, number of parathyroid gland detected on color ultrasound, number of parathyroid gland detected on ECT, number of parathyroid gland detected through intraoperative pathology, preoperative BNP, serum Ca, serum P, TSH and AAC score were not significant upon logistic analysis (*P* > 0.05) (Tables 2, 3).

**Table 2:** Univariate logistic regression analysis on the risk of HBS after t-PTX

Predictor	*β*	SE	EXP(*β*)	95% CI	χ^2^	*P* value
Female	−0.265	0.495	0.767	0.291–2.024	0.289	0.592
Hemodialysis	−0.304	1.434	0.738	0.044–12.264	0.045	0.832
Hypertension	1.406	1.122	4.079	0.452–36.769	2.003	0.210
Diabetes	0.752	0.945	2.121	0.333–13.505	0.650	0.426
Age (years)	−0.032	0.22	0.968	0.927–1.011	2.180	0.147
Duration of dialysis (years)	0.054	0.067	1.055	0.925–1.204	0.644	0.425
BMI (kg/m^2^)	−0.079	0.069	0.924	0.807–1.058	1.403	0.253
Frequency of color ultrasound	0.314	0.249	1.369	0.841–2.229	1.683	0.206
Frequency of ECT	−0.106	0.320	0.900	0.48–1.686	0.109	0.741
Frequency of pathological examination	0.308	0.676	1.361	0.362–5.116	0.212	0.648
Preoperative Hb (g/l)	−0.050	0.016	0.951	0.922–0.981	12.588	0.002
BNP (pg/ml)	0.001	0.000	1.001	1.000–1.002	1.534	0.233
Alb (g/l)	−0.146	0.067	0.864	0.758–0.986	5.593	0.030
ALP (U/l)	0.010	0.002	1.010	1.005–1.014	48.763	0.000
Serum calcium (mmol/l)	−1.564	0.962	0.209	0.032–1.380	2.809	0.104
Serum phosphate (mmol/l)	−0.063	0.442	0.939	0.395–2.233	0.021	0.886
iPTH (pg/ml)	0.002	0.000	1.002	1.001–1.003	37.359	0.000
TSH (mlu/l)	−0.159	0.191	0.853	0.586–1.242	0.719	0.407
AAC score	0.019	0.028	1.019	0.965–1.077	0.482	0.488

**Table 3:** Multivariate logistic regression analysis on the risk of HBS after t-PTX

Predictor	*β*	SE	EXP(*β*)	95% CI	*P* value
Preoperative Hb (g/l)	−0.019	0.027	0.981	0.930–1.034	0.476
Alb (g/l)	−0.079	0.123	0.924	0.726–1.174	0.517
Serum ALP/10 (U/l)	0.095	0.030	1.100	1.037–1.166	0.001
Preoperative iPTH/100 (pg/ml)	0.105	0.048	1.111	1.011–1.220	0.029
Constant	−0.253	4.311	0.777		0.953

### Multivariate logistic regression analysis on the incidence of HBS after t-PTX

**Fig. 1:**
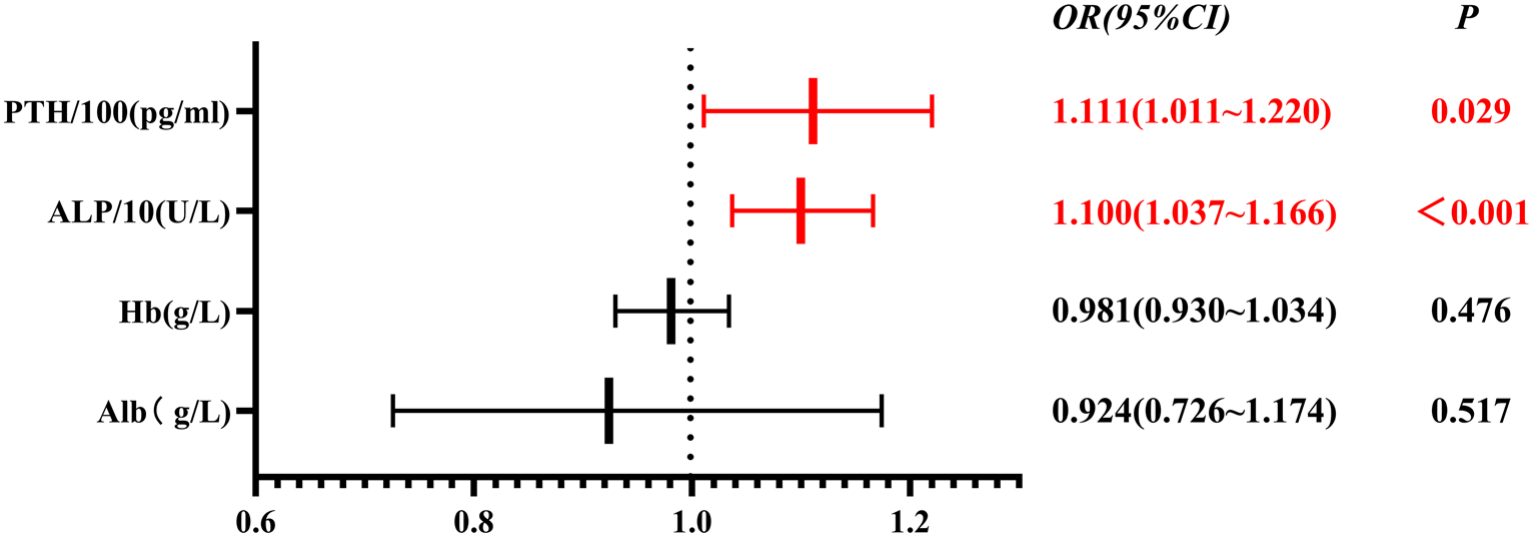
Multivariate logistic regression analysis on the risk of HBS after t-PTX

Based on the above-mentioned univariate analysis results, the preoperative Hb, Alb, iPTH and ALP were selected for multivariate logistic regression analysis (Fig. 1). As ALP and PTH were the continuous variables with great value ranges, the change in one unit of measurement made little influence on the outcome. Therefore, iPTH /100 and ALP/10 were selected as the variables for analysis. The results indicated that preoperative iPTH level (OR = 1.111, *P* = 0.029) and ALP level (OR = 1.010, *P* < 0.001) were the independent risk factors for postoperative HBS.

### Construction of the logistic regression model and the model calibration

Based on the above-mentioned logistic regression analysis, the prediction model of the HBS risk after t-PTX was constructed as follows: [Logit (*P*) = −0.253 + 0.0095 × (ALP) + 0.00105 × (iPTH)]. The Hosmer–Lemeshow testing model showed a high degree of fitting with the observed values (χ^2^ = 3.405, *P* = 0.474).

### Prediction performance of the logistic regression model

**Fig. 2:**
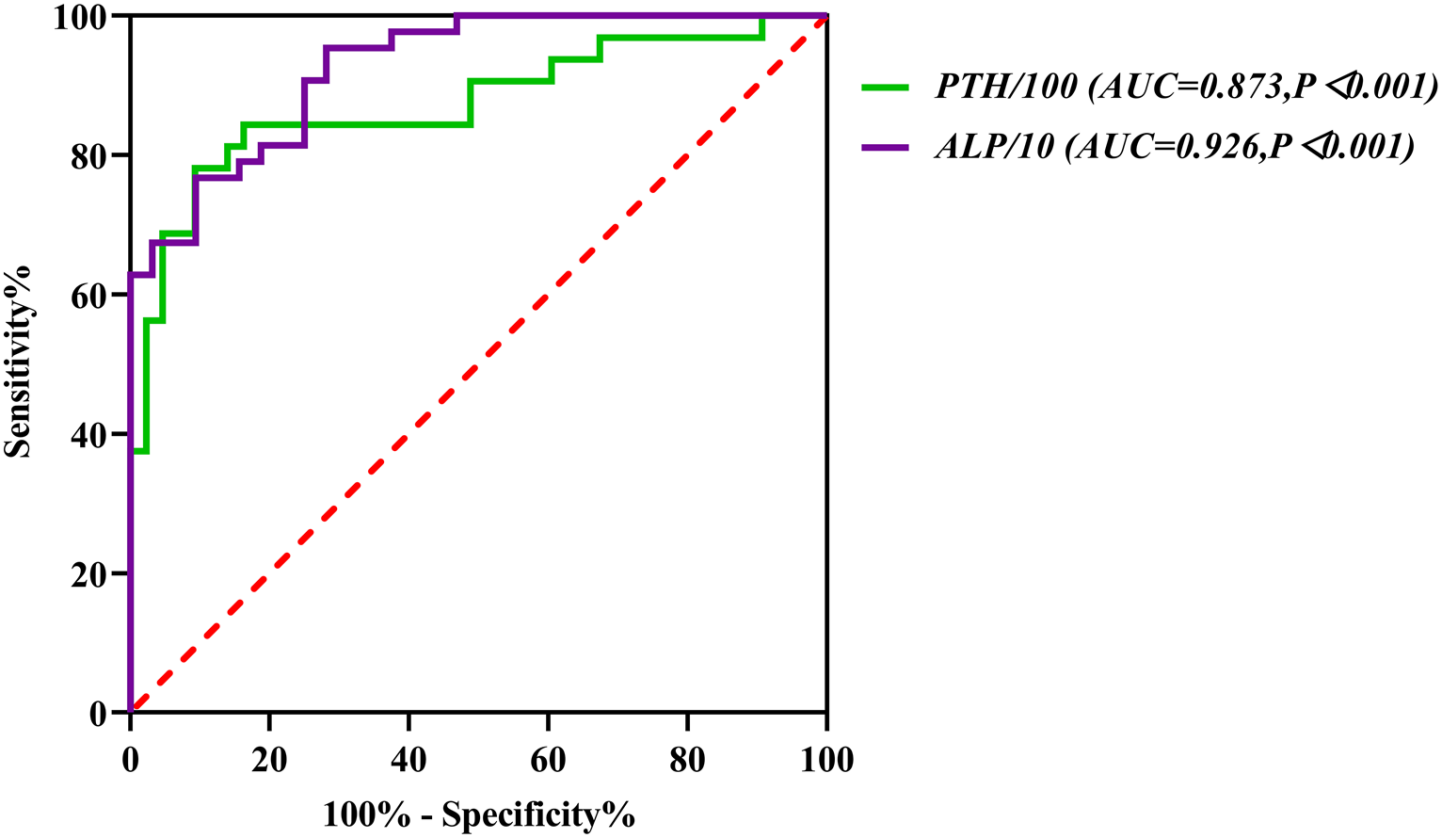
ROC curve of the prediction model for HBS after t-PTX

The ROC curves were plotted to analyze the model prediction performance. The AUC values of the risk of postoperative HBS predicted by the model were shown below. The AUC value of preoperative ALP was 0.926 (95% CI 0.871–0.980) (*P* < 0.0001) and that of preoperative iPTH was 0.873 (95% CI 0.785–0.961) (*P* < 0.0001). Both achieved high prediction values for preoperative HBS (Fig. 2), and their respective optimal cutoff values (namely, Youden index, YI) were 2433.1 pg/m and 289.5 U/l, respectively (Table 4).

**Table 4:** Cutoff values of the prediction model for HBS after t-PTX

	Sensitivity	Specificity	Standard error	95% CI	Cutoff	AUC	*P*
ALP (U/l)	0.906	0.767	0.028	0.871–0.980	289.5	0.926	< 0.001
iPTH (pg/ml)	0.781	0.907	0.045	0.785–0.961	2433.1	0.873	< 0.001

### Nomogram model

**Fig. 3:**
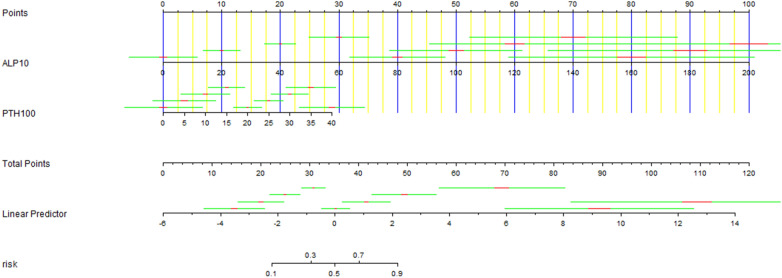
Nomogram showing the risk of HBS after t-PTX

**Fig. 4:**
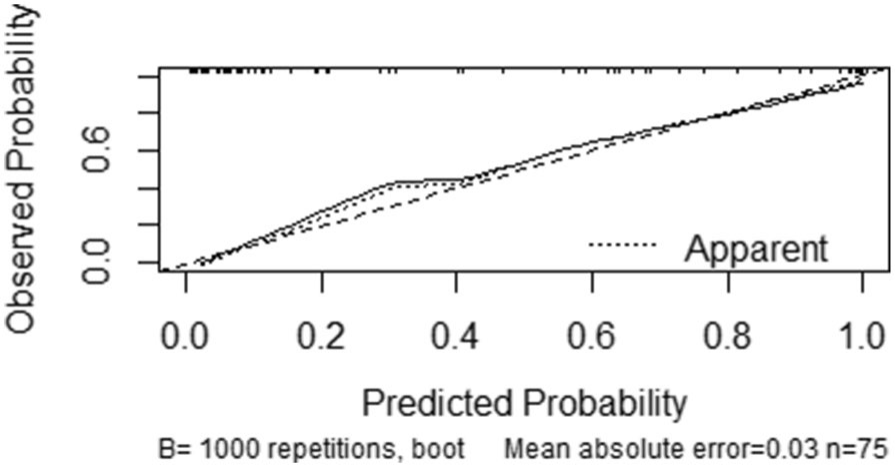
Calibration curve of the prediction model for HBS after t-PTX

Risk factors were selected based on multivariate logistic regression analysis. Afterwards, data were imported into the R software to construct the nomogram model for the prediction of HBS incidence after PTX (Fig. 3). Then, the Bootstrap adaptive sampling method was used for internal verification of the model. After repeated sampling for 1000 iterations, the C-index was calculated to be 0.943 (95% CI 0.892–0.994). The calibration curve showed a good degree of fitting with the ideal curve, suggesting the favorable degree of fitting of the model (*P* < 0.0001) (Fig. 4).

## Discussion

Our research results demonstrated that, high serum ALP and high serum iPTH levels preoperatively were the independent risk factors for HBS after PTX.

There are numerous approaches for PTX, including the most common t-PTX, subtotal PTX, t-TPX + forearm transplantation, and color ultrasound-guided radiofrequency ablation (RFA) [17]. In this study, all patients selected received t-PTX, which more truly reflected the postoperative HBS levels. For patients undergoing subtotal PTX or forearm transplantation, the probability of postoperative HBS is certainly reduced due to the partial residual parathyroid function, which will interfere with the real HBS illness.

Currently, PTH is the most common index used to evaluate the severity of CKD-MBD. With the gradual decline of renal function in ESKD patients, the P scavenging effect of renal tubule also decreases accordingly; meanwhile, inadequate active vitamin D is produced to maintain the normal serum Ca content. To maintain the homeostasis of serum Ca and P levels, the expression and release of iPTH tend to increase. When excessive iPTH is released in the body and exceeds the physiological requirement, the balance between bone formation and bone resorption is broken, and the effect of the abnormally elevated PTH level on osteoblasts and osteocytes indirectly increase the number and activity of osteoclasts [15, 18–20]. The enhanced bone resorption of osteoclasts can lead to osteolysis, and excess minerals (like Ca and P) are released into the blood, which can be manifested as the elevated serum bone turnover index if they cannot be timely eliminated by the kidney [17]. When the iPTH level rapidly declines after PTX, the osteoclast activity in the body decreases, the osteolysis stops, the osteoblast activity increases, and a large amount of circulating Ca ions are transferred to the bone to participate in bone formation; thus, the serum Ca level rapidly declines after PTX, as demonstrated in studies in vitro [21–24]. This study proved that the high preoperative iPTH level was a risk factor for postoperative HBS. Hopefully, clinicians can realize the vital prediction value of preoperative iPTH, avoid the occurrence of HBS and maximally reduce the adverse consequences through better understanding the postoperative course of disease and improving the perioperative management.

The ALP activity is of crucial importance to the appropriate bone mineralization. ALP is also the well-recognized biomarker for renal osteodystrophy [25–27]. SHPT patients are mostly associated with metabolic diseases, their serum ALP levels increase in the presence of activated osteoblasts, and the increase level is associated with the severity of bone disease [17, 28]. The preoperative ALP concentrations are high in HBS patients, revealing the more active bone remodeling status in HBS patients after PTX [29, 30]. Based on this study, preoperative ALP level was the risk factor for postoperative HBS. ALP level can guide clinicians to recognize the dialysis patients who are at a higher risk of HBS after PTX, monitor and treat the high-risk patients postoperatively; for instance, to construct the central venous catheter immediately after surgery, and actively supplement Ca and active vitamin D analogues.

Compared with other clinical studies, this study has certain strengths. At first, from the perspective of methodology, this study constructed the nomogram based on the traditional logistic regression analysis prediction formula, and transformed the complex regression equation into the visual diagram, so that the prediction model results were more readable, making it convenient for patient evaluation. Secondly, the surgical method in this study was unified, in other words, all the enrolled patients received the same surgical procedure (t-PTX), effectively avoiding the difference in HBS occurrence probability due to different surgical procedures. Nevertheless, several limitations should be noted in this study. Firstly, it was a retrospective, observational study, and thus the results might be associated with selection bias. Secondly, the sample size in this study was relatively small, and the potentially significant association between variables might be covered. Thirdly, this study did not monitor the potential risk factor indexes of HBS mentioned in other studies, including 1,25 (OH)_2_D level, bone-ALP level, osteocalcin, and weight of the resected parathyroid gland, which might have certain influence on the results [31, 32].

## Conclusion

To conclude, postoperative HBS is extremely rare and dangerous, but it is one of the signs of successful surgery and also the chance to repair skeletal lesion of the patient, which deserves attention from clinicians. This study analyzed the clinical data of 75 patients undergoing PTX in our center. Our results suggested that preoperative ALP and iPTH were the independent risk factors for HBS after PTX. The corresponding optimal cutoff values were obtained through ROC curve analysis. The construction of nomogram model helps clinicians to carry out early intervention on patients. For high-risk patients, the frequency of serum Ca monitoring should increase early after surgery. Importantly, in the early stage of hypocalcemia, active Ca supplementation is recommended to reduce the incidence of severe HBS.

## Data Availability

All the datasets are available from the corresponding author on reasonable request.

## Abbreviations

ESKD: End-stage kidney disease
IPTH: Intact parathyroid hormone
SHPT: Secondary hyperparathyroidism
PTX: Parathyroidectomy
HBS: Hungry bone syndrome
N-HBS: Non-hungry bone syndrome
ROC: Receiver operating characteristic curve
HD: Hemodialysis
PD: Peritoneal dialysis
ALP: Alkaline phosphatase
ORs: Odds ratios
95%CI: 95% Confidence intervals
CKD: Chronic kidney disease
AUC: Area under curve
AAC: Abdominal aortic calcification
Ca: Calcium
P: Phosphorus
Alb: Albumin
Hb: Hemoglobin
TSH: Thyroid stimulating hormone
t-PTX: Total parathyroidectomy
